# Effect of artificial toothbrushing on wear, surface roughness and hardness of additively manufactured and milled resin-based restorative materials: an in vitro study

**DOI:** 10.1186/s12903-026-08406-3

**Published:** 2026-05-06

**Authors:** Yasser M. Aly, Mohamed Mahmoud El-Kateb, Nodar Mohamed Ghrebi

**Affiliations:** 1 Consevative Dentistry Department, Faculty of Dentistry, Alexandria University, Champollion Street, Azarita, Alexandria, Egypt; 2 Oral Rehabilitation Sciences, Faculty of Dentistry, Beirut Arab University, Beirut, Lebanon

**Keywords:** Wear, Flexcera, BRILLIANT crios, Vickers hardness number (VHN), CROWNTEC

## Abstract

**Background:**

Recently introduced milled and 3D-printed resin dental restorations are designed for long-term use. They must endure the mechanical stresses of chewing in the oral environment. This study aimed to evaluate the effect of artificial toothbrushing on wear, surface roughness and hardness of additive-manufactured and milled ceramic filled resin restorative materials.

**Materials and methods:**

Thirty bar-shaped specimens (15 mm x 4 mm x 1.5 mm) were divided into three groups (*n* = 10): 3D printed CROWNTEC Saremco permanent resin (CT), 3D printed Flexcera Smile Ultra+ (FSU), and CAD-CAM BRILLIANT Crios (BC). All groups underwent the same finishing protocol using a composite polishing kit. The specimens baseline weight was first measured using a sensitive electronic balance, then tested for surface microhardness using the Vickers hardness test (VHN), and for surface roughness using confocal laser scanning microscopy (CLSM). Wear testing was conducted using an artificial toothbrushing machine. Specimen wear was calculated by evaluating weight loss. The Vickers hardness test and surface roughness test were then repeated. Differences in wear and hardness were analyzed using one-way ANOVA followed by Tukey’s post hoc test with Bonferroni correction. Changes before and after artificial toothbrushing were assessed using a paired *t*-test. The Kruskal–Wallis test, followed by Dunn’s post hoc test with Bonferroni adjustment, was employed to analyze surface roughness values. The Wilcoxon signed-rank test was used to assess changes in surface roughness after toothbrushing.

**Results:**

Flexcera Smile Ultra+ demonstrated the least wear reduction, CROWNTEC the middle, and BRILLIANT Crios the most. All groups had significant change in hardness before and after intervention. BRILLIANT Crios changed least, CROWNTEC somewhat, and Flexcera Smile Ultra+ most. Using One Way Analysis of Variance ANOVA, significant wear and hardness differences were found between groups before and after the intervention (p < 0.0001). The Kruskal-Wallis test showed significant surface roughness changes between groups before and after intervention (p < 0.0001). The intervention reduced surface roughness differently across materials, with Flexcera Smile Ultra+ showing the largest consistent reduction.

**Conclusions:**

All materials that were tested exhibited notable variations in terms of wear, hardness, and surface roughness both prior to and following an artificial toothbrushing, which depended on their respective qualities. Regarding wear, FSU exhibited the least reduction, CT showed a moderate reduction, and BC experienced the most significant reduction. In terms of hardness, BC underwent the least change, CT displayed a moderate change, while FSU experienced the greatest change. Milled resin was found to have the lowest surface roughness, whereas 3D printed resin demonstrated the highest surface roughness.

## Background

The increasing demand for dental treatments that are both aesthetic pleasing and durable has driven the development of advanced restorative options, particularly in indirect adhesive restorations. Technologies like CAD/CAM and 3D printing have played a key role in this progress. Recent advancements in resin-based composites have enhanced the efficiency of CAD/CAM systems in producing inlays, onlays, crowns, and bridges [1].

Simultaneously, 3D printing has emerged as a transformative tool in dentistry, providing precise and customizable solutions. However, additive manufacturing is becoming a valuable alternative to traditional subtractive methods offering accessibility and versatility for various dental applications [2–6].

Recently, additive manufacturing using 3D printed resin, with or without ceramic particles, has been marketed as a definitive restoration solution. Parameters such as filler content, processing technique, curing mode, and layer thickness may influence the surface properties and qualities of the final restorations [1].

Digital light processing (DLP) employs a stationary digital micromirror apparatus to reflect and concentrate ultraviolet (UV) light, thereby facilitating the curing of an entire resin layer simultaneously. The process of layer curing in DLP differs from that of SLA [7].

This study involved three resin materials: two 3D-printed resins and one CAD/CAM composite block. BRILLIANT Crios (Coltene) which is a reinforced composite containing barium glass and silica particles in a methacrylate matrix (70.7% filler by weight). With an elastic modulus like dentin, it helps in reducing stress and prevent fractures, making it suitable for onlays and overlays. Though clinical data on CAD/CAM composites is limited, reported 10-year survival rates reach 95% [8]. CROWNTEC (CT) is a light-cured composite resin fabricated via additive manufacturing [9]. Flexcera Smile Ultra+ (FSU) is engineered for superior moisture resistance, fracture toughness, and aesthetics, incorporating ceramic nanoparticles and a blend of acrylates, methacrylates, and fillers [10].

Wear resistance is a key mechanical property in restorative dentistry, as it serves as a predictor of the material’s long-term performance and clinical durability [11]. Surface roughness is closely associated with wear behavior; increased roughness often indicates material degradation. Therefore, evaluating surface roughness through in-vitro wear simulation, such as artificial toothbrushing, is a valuable method for assessing the durability of restorative materials [12].

Confocal Laser Scanning Microscopy (CLSM), a reflection-based imaging technique, has recently gained attention in dental research for its ability to provide high-resolution, three-dimensional surface characterization. Although its use remains limited compared to fluorescence microscopy, CLSM offers precise analysis of surface topography [13–15].

The Vickers hardness test, commonly used in dental material evaluation, provides reliable measurements independent of indenter size, using a diamond pyramid-shaped indenter applicable across a wide range of materials. The resulting hardness values are reported as Vickers Pyramid Number (HV) or Diamond Pyramid Hardness (DPH) [16].

Despite the increasing adoption of 3D printing in dental applications, data on the effects of simulated toothbrushing on the mechanical properties of 3D-printed restorative materials particularly those intended for definitive restorations remain scarce. This in-vitro study aimed to assess the surface roughness and microhardness of CAD-CAM restorative materials both 3D printed and milled following simulated wear through artificial toothbrushing, which are critical factors in the functional longevity of permanent restorations.

The investigation tested several null hypotheses, primarily that no statistically significant differences would be observed in wear resistance, surface roughness, or microhardness before and after brushing under increased load conditions, irrespective of the manufacturing technique used.

## Materials and methods

The sample size was determined based on data from a previous study [17] using statistical software (G*Power v3.1.9.7; Heinrich-Heine-Universität Düsseldorf) ) [18]. The calculation was performed with a significance level (α) of 0.05 and a statistical power of 80%, resulting in a minimum required sample size of nine specimens per group. To account for potential processing errors, the sample size was increased to ten specimens per group, leading to a total sample size of 30 specimens. All specimens were used to assess wear, surface roughness, and microhardness using statistical analysis software (IBM SPSS, Version 23; Armonk, NY, USA).

**Fig. 1:**
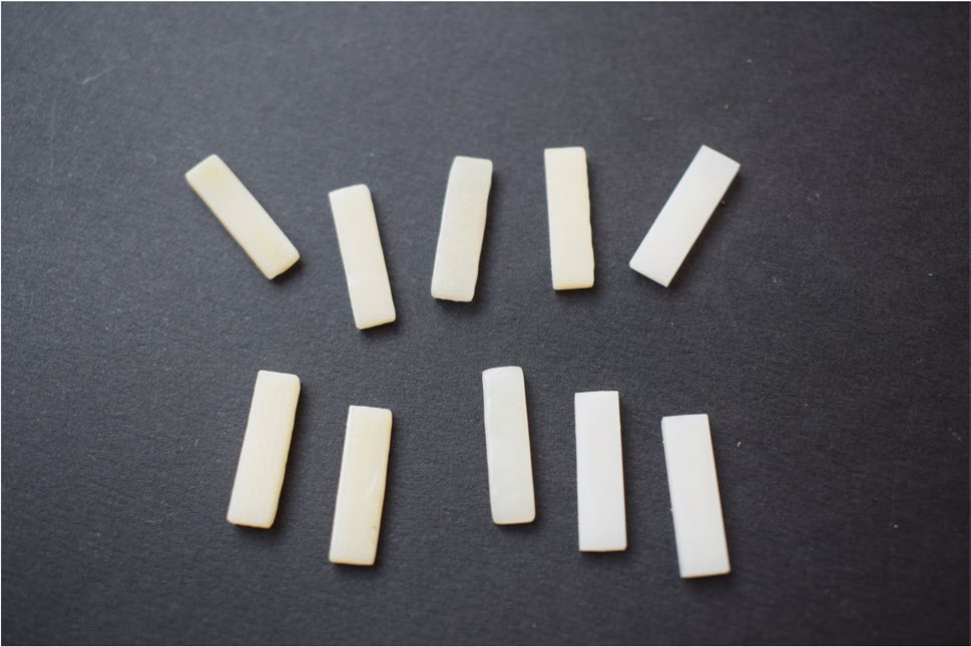
3D printed and milled specimens utilized in the study

**Fig. 2:**
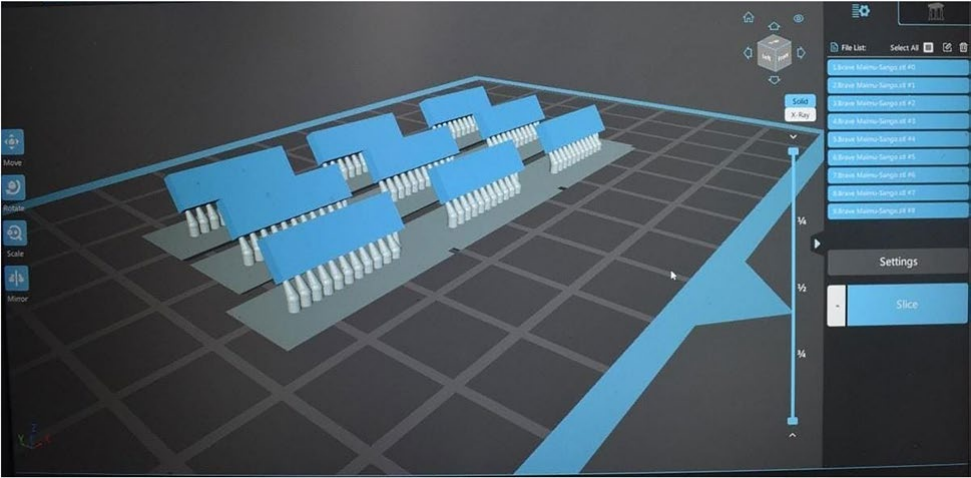
Pre-processing the 3D printed (CT&FSU) specimens using a 3D printer before starting the printing process

A rectangular model measuring 15 × 4 × 1.5 mm was created in STL format using Blender software (version 3.2, Blender Institute, Netherlands). This design was used to produce 30 specimens divided into three groups depending on the material used **(**Fig. 1**)**. Among these, 10 specimens were subtractively manufactured using (Brilliant Crios; Coltene [BC] CAD-CAM material, while 20 specimens were additively manufactured using (Crowntec; Saremco Dental AG [CT] (*n* = 10) and Flexcera Smile Ultra+; Envision TEC GmbH [FSU]) (*n* = 10)) as in **(**Fig. 2**)**. Detailed information and abbreviations of the test groups are listed in Table 1. 

**Table 1:** Brand, manufacturer, and composition materials were used in this study

Product	Composition	Manufacturer	Lot. number
BRILLIANT Crios,	70.7% <20 nm Amorphous silica and < 1 μm barium glass. -29.3% Cross-linked methacrylate resin matrix (Bis-GMA, Bis-EMA, TEGDMA) (SiO, < 20 nm, barium glass < 1 μm forming 70.7 wt% inorganic filler 10.3 GPa	COLTENE, Switzerland	K25739
CROWNTEC	Esterification products of 4,4’-isopropylidiphenol, ethoxylated and 2-methylprop-2enoic acid, silanized dental glass, Pyrogenic silica, initiators. Total content of inorganic fillers (particle size 0.7 μm) is 30–50% by mass.	Saremco dental, Switzerland	E622
Flexcera Smile Ultra+,	Acrylates, methylacrylates, methacrylated oligomers and monomers, photo initiators, colorants/dyes, fillers and absorbers.	Desktop Health, Germany	310122b

The additively manufactured specimens were fabricated with a Digital Light Processing (DLP) 3D printer (Phrozen Sonic Mini 4 K, Taiwan) at a layer resolution of 50 μm and in a vertical orientation on the platform. Following fabrication, the 3D-printed specimens underwent a two-step cleaning protocol in accordance with the manufacturer’s instructions, utilizing 96% ethanol and an unheated ultrasonic bath (Codyson CD-4820, Shenzhen, China). Initially, the specimens were immersed in a reusable ethanol solution for 3 min, followed by a second immersion in fresh ethanol for 2 min to enhance resin removal. After ultrasonic cleaning, the specimens were rinsed with an additional 96% ethanol to eliminate any remaining uncured resin residues [6].

They were then dried using compressed air under an extraction system. To optimize mechanical performance, ensure complete polymerization, and minimize residual monomer content, the specimens underwent post-curing in an ultraviolet (UV) light-curing unit (Brelux Power Unit 2, Chesterfield, UK), and were subsequently cooled for 3–5 min. Finally, all supporting structures of the final printed products were removed using a cutting wheel.

The subtractively manufactured bars specimens (15 mm × 4 mm × 1.5 mm) were sectioned from BRILLIANT Crios CAD/CAM blocks (Coltène, Whaledent A.G., Altstätten, Switzerland) using a precision cutting machine (Isomet 4000, Buehler Ltd., USA) equipped with a water-cooled diamond blade (0.6 mm thickness; Buehler, USA). Following sectioning, the specimens were further finished using wet silicon carbide abrasive paper (grit 400, ISO/FEPA standard, average particle size 35 μm) to ensure uniform surface quality.

All specimens were measured with a digital caliper (IP54-, Qfun, China) to ensure their final dimensions and stored in a dry condition at room temperature [15].

**Fig. 3:**
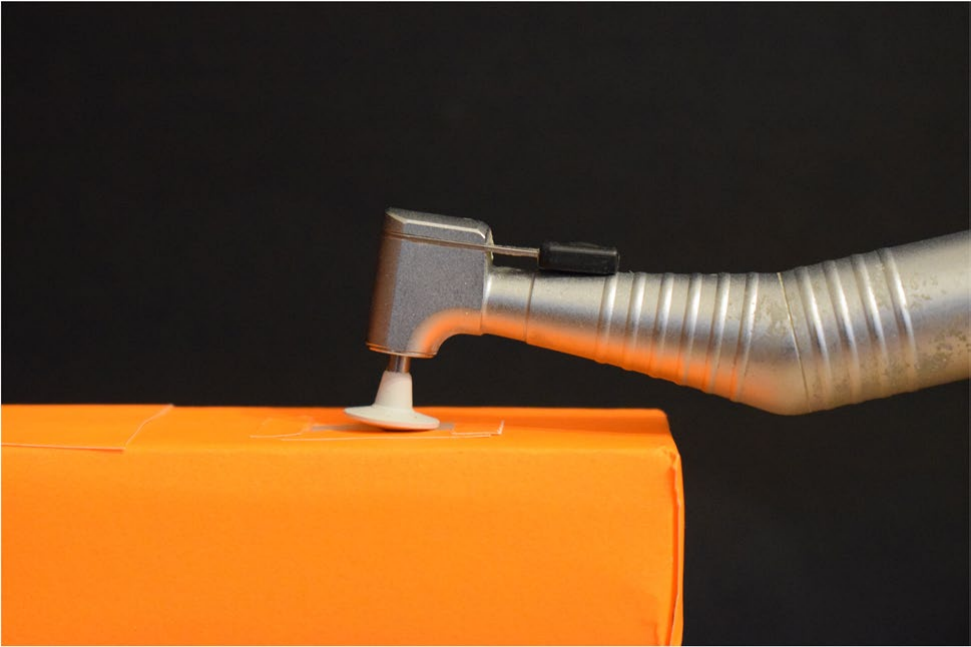
A custom-made rectangular cubic Teflon mold to secure the specimen throughout the polishing process

The specimens were then polished using a composite polishing system (EVE Composoft, Keltern, Germany) attached to a contra-angle low-speed handpiece (W&H, Bürmoos, Austria) driven by a micromotor. The objective of the polishing procedure was to produce a uniformly smooth surface across all specimens. A custom-made rectangular Teflon mold (15 cm × 3 cm × 2 cm) was fabricated from a Teflon blank, featuring two central rectangular housings (15 mm × 4 mm × 1.5 mm) **(**Fig. 3**)** corresponding to the specimen dimensions. These housings were designed to securely hold the specimens during the polishing process [17].

The polishing burs were mounted on the angled handpiece, which was stabilized using a metal holder (22 cm × 15 cm × 3 cm) fixed to a wooden base to ensure consistent positioning and distance throughout the procedure. Following polishing, all specimens were subjected to ultrasonic cleaning (Codyson CD-4820, Shenzhen, China) in 96% ethanol to remove surface debris. After cleaning, the specimens were dried using absorbent paper and air, then prepared for subsequent weighting [18].

The specimens were stored in distilled water at 37.5 °C for one week in an incubator to mimic and maintain the optimal temperature of the mouth [19]. Prior to the artificial toothbrushing procedure, each specimen was weighed using a high-precision electronic analytical balance (RADWAG, Radom, Poland).

Baseline measurements of microhardness (VHN) and surface roughness were recorded prior to simulated tooth brushing testing (wear test).

The wear simulation test was performed using a custom-designed toothbrushing simulation device. Mechanical brushing was applied for 10,000 cycles to replicate approximately one year of clinical use [20]. A vertical load of 2.0 N (equivalent to 200 g) was applied using a manual toothbrush (Colgate Twister; Colgate-Palmolive, São Paulo, Brazil). The abrasive slurry was prepared according to ISO 14569-1:2007 by mixing 250 g of toothpaste (Colgate Total; RDA = 70) with 1 L of distilled water, and it was refreshed every 5,000 brushing cycles.

Upon completion of the brushing protocol, specimens were ultrasonically cleaned (Codyson CD-4820, Shenzhen, China), rinsed with distilled water, and dried. Quantitative wear was assessed by measuring weight loss before and after brushing using a high-precision electronic analytical balance (RADWAG, Radom, Poland) [21].

The extent of wear was expressed as the change in mass:

- Mean weight loss = Initial weight (W₁) – Final weight (Wf)
- Percentage of weight loss = [(W₁ – Wf) / W₁] × 100

Where:

- W₁ = weight before the wear test (mg)
- Wf = weight after the wear test (mg) [22]

Following the completion of the wear test, surface roughness and Vickers Hardness Number (VHN) measurements were re-evaluated to assess the post-wear changes in the specimens.

The Vickers Hardness Number (VHN) was measured before and after the artificial toothbrushing test using a microhardness tester (HST-1000-A, West, Jinan, China) in accordance with ISO 14233:2003 standards. Each experimental group included five specimens (total *n* = 15). For each specimen, three indentations were made, spaced approximately 3 mm apart. VHN was determined by measuring the average lengths of the two diagonals formed by each indentation.

Indentations were performed using a diamond pyramid-shaped indenter with a 136° angle between opposing faces, applying a load of 1.961 N for 15 s. The mean value of the three indentations per specimen was recorded as the final VHN.

The Vickers Hardness (H) was calculated using the following formula: H = 1.961×F/D²,

where:

- F is the applied load in newtons (N),
- D is the mean diagonal length of the indentation in millimeters (mm²) [9].

All the tested specimens were subjected to surface roughness measurement before and after the wear test. Samples from all groups were individually positioned on the stage of a CLSM (VK-X100, KeyenceGmbH, Neu-Isenbuerg, Germany) with the machined surface facing upward and then scanned. Using an Ar/Ar Kr laser (Emission 488 nm blue, 568 nm green, 647 nm red), a reflection image of the surface was produced [23].

The subsequent roughness parameters were assessed for each of the 3 regions of interest.

A roughness profile was generated for the region of interest to include surface area, average height/roughness (Ra). Diagrammatic representation of a scan carried out using CLSM [23].

### Statistical analysis

Normality was checked using *Shapiro Wilk test* and *Q-Q plots.* Wear and Hardness were normally distributed. Data values were summarized using mean, standard deviation (SD), minimum and maximum values.

Differences in wear and hardness were analyzed using *One Way ANOVA* and followed by *Tukey’s post hoc test* with Bonferroni correction while changes before and after artificial toothbrushing were assessed using *Paired t test*. *Kruskal Wallis* test with *Dunn’s post hoc test* with Bonferroni adjustment was employed to analyze surface roughness values. *Wilcoxon Sign Rank test* was used to assess changes in surface roughness after toothbrushing. All tests were two tailed and the significance level was set at p value < 0.05. IBM SPSS version 23, for Windows, Armonk, NY, USA was used for data analysis.

## Results

### Wear test

The study assessed the baseline weight of the CT, BC, and FSU groups before the intervention, as well as the wear experienced after the intervention (Table 2). The mean ± SD baseline weight before intervention was 0.136 ± 0.009 for CT, 0.192 ± 0.005 for BC, and 0.114 ± 0.007 for FSU. Post-intervention, mean ± SD wear values declined to 0.135 ± 0.010 for CT, 0.189 ± 0.005 for BC, and 0.113 ± 0.007 for FSU. Using One Way Analysis of Variance ANOVA, significant wear differences were found between groups before and after the intervention (*p* < 0.0001).

**Table 2:** Comparison between the baseline weight before the intervention and the wear exhibited after the intervention among the three groups

		CROWNTEC Saremco (*n* = 10)	BRILLIANT Crios (*n* = 10)	Flexcera Smile Ultra+ (*n* = 10)	Test^1^ (*p* value)
Before	Mean ± SD	0.136 ± 0.009	0.192 ± 0.005	0.114 ± 0.007	301.424 (< 0.0001*)
	Median	0.131	0.194	0.116	
	Min – Max	0.127–0.150	0.182–0.197	0.102–0.120	
After	Mean ± SD	0.135 ± 0.010	0.189 ± 0.005	0.113 ± 0.007	282.409 (< 0.0001*)
	Median	0.130	0.192	0.115	
	Min – Max	0.126–0.149	0.180–0.195	0.101–0.119	
Test^2^ (*p* value)		7.261 (< 0.0001*)	3.621 (0.006*)	10.500 (< 0.0001*)	

Test1: One Way Analysis of Variance, Test2: Paired t

*Statistically significant difference at p value < 0.05

Additionally, paired t tests indicated significant reductions in wear within each group following the intervention. These findings highlighted that all materials experienced a reduction in wear following the intervention. (Table 5)

### Hardness test

**Fig. 4:**
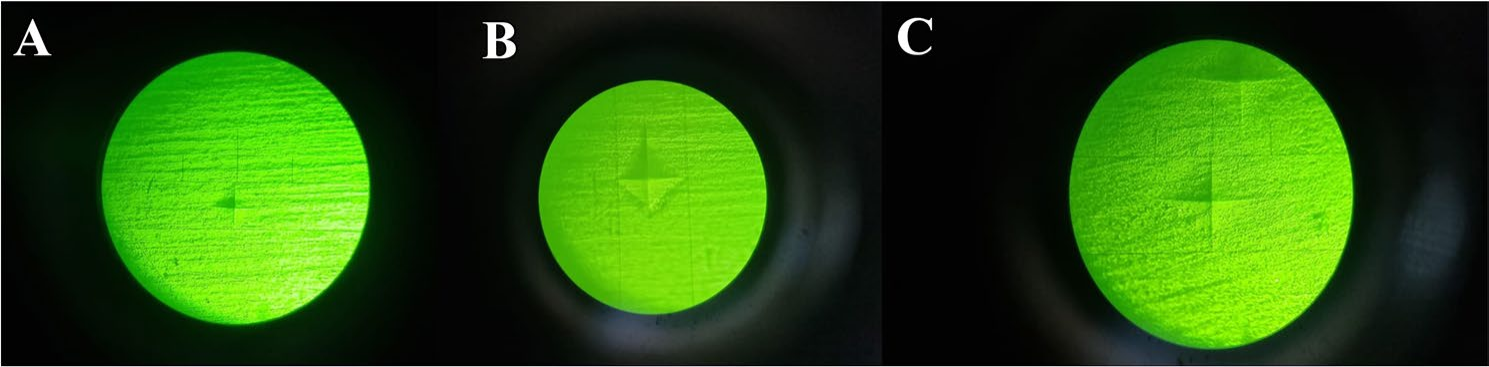
Showing the hardness indentation marks before simulated tooth brushing (wear test), **A** for BC, **B** for FSU, and **C** for CT

**Fig. 5:**
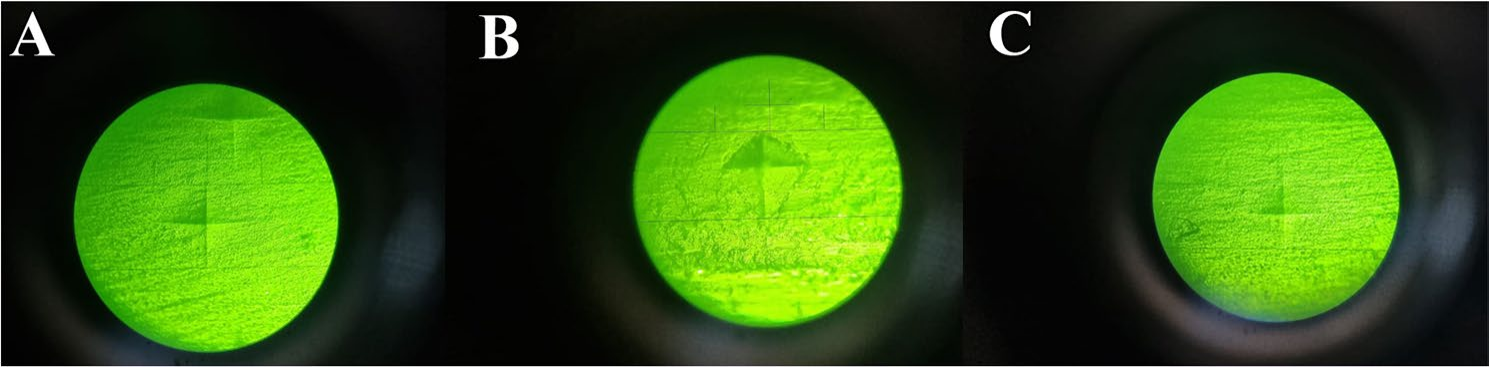
Showing the hardness indentation marks after simulated tooth brushing (wear test), **A** for BC, **B** for FSU, and **C** for CT

Regarding the tested groups, before intervention, CT had a mean hardness of 34.07 ± 3.74, BC had 71.43 ± 4.08, and FSU had 16.73 ± 0.70 **(**Fig. 4**)**. After intervention, these values changed significantly, with CT reduced to 25.00 ± 3.12, BC to 59.10 ± 3.08, and FSU to 12.20 ± 1.74 **(**Fig. 5**).** Statistical analyses using One-way ANOVA (Test1) revealed significant differences in hardness between groups before and after intervention (*p* < 0.0001). Paired t tests (Test2) revealed significant decrease in hardness within each group post-intervention of the study groups (*p* < 0.0001) (Tables 3 and 5).

**Table 3:** Comparison of hardness among the study groups before and after intervention

		CROWNTEC Saremco (*n* = 10)	BRILLIANT Crios (*n* = 10)	Flexcera Smile Ultra+ (*n* = 10)	Test^1^ (*p* value)
Before	Mean ± SD	34.07 ± 3.74	71.43 ± 4.08	16.73 ± 0.70	679.044 (< 0.0001*)
	Median	32.20	74.00	16.60	
	Min – Max	31.00–39.00	66.00–74.30	16.00–17.00	
After	Mean ± SD	25.00 ± 3.12	59.10 ± 3.08	12.20 ± 1.74	713.336 (< 0.0001*)
	Median	26.00	61.00	12.00	
	Min – Max	21.00–28.00	55.00–61.30	10.30–14.30	
Test^2^ (*p* value)		7.021 (< 0.0001*)	37.00 (< 0.0001*)	13.071 (< 0.0001*)	

Test1: One Way Analysis of Variance, Test2: Paired t test

*Statistically significant difference at p value < 0.05

The material with the least amount of change was BC, while CT exhibited a moderate shift, and FSU showed the highest level of change.

### Surface roughness test

**Fig. 6:**
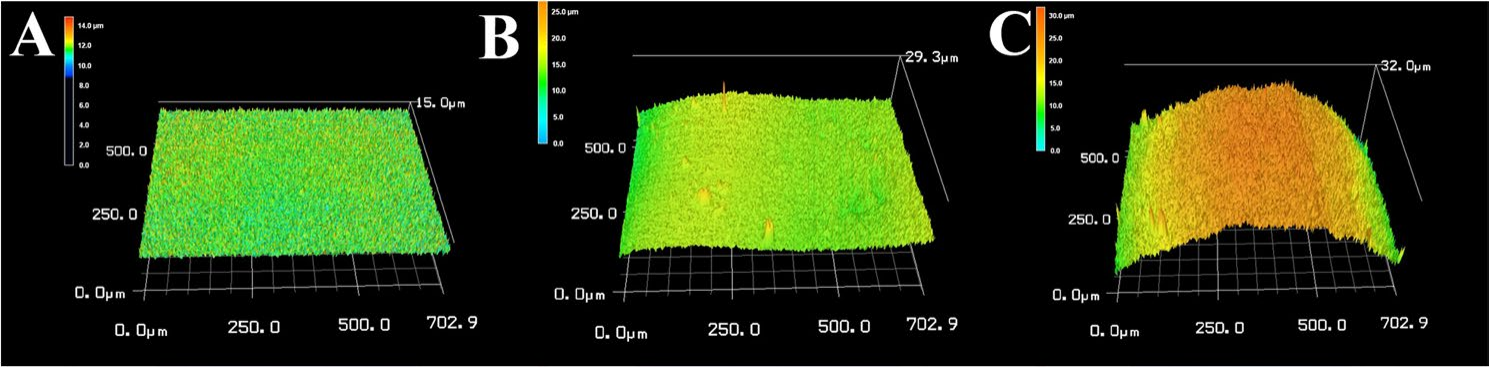
CLSM images for the three tested materials before simulated tooth brushing. **A** for BC, **B** for FSU, and **C** for CT, BC showed the more homogenous flat surface

**Fig. 7:**
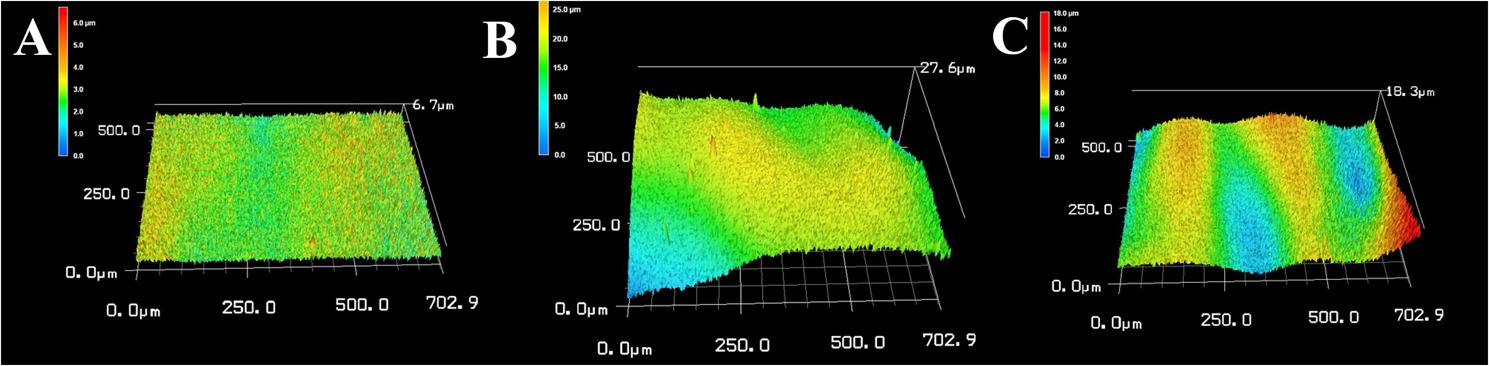
CLSM images of the three tested materials after simulated tooth brushing: **A **for BC, **B **for FSU, and **C **for CT. Roughness increased in all materials, although BC remained more regular in surface texture

Regarding the tested groups, before intervention, CT exhibited a median surface roughness of 1.08 (range: 0.66 to 1.13), BC had a median of 0.49 (range: 0.45 to 0.49), FSU showed a median of 0.94 (range: 0.68 to 1.31) **(**Fig. 6**)**. After intervention, these values changed to a median surface roughness of 1.52 (range: 0.69 to 2.68) for CT, 0.52 (range: 0.40 to 0.53) for BC, and 1.58 (range: 1.38 to 1.71) for FSU (Table 4) **(**Fig. 7**)**. The Kruskal-Wallis test showed significant surface roughness changes between groups before and after intervention (*p* < 0.0001) (Table 5).

**Table 4:** Comparison of surface roughness among the study groups before and after intervention

		CROWNTEC Saremco (*n* = 10)	BRILLIANT Crios (*n* = 10)	Flexcera Smile Ultra+ (*n* = 10)	Test^1^ (*p* value)
Before	Mean ± SD	0.96 ± 0.22	0.47 ± 0.02	0.98 ± 0.27	17.622 (< 0.0001*)
	Median	1.08	0.49	0.94	
	Min – Max	0.66–1.13	0.45–0.49	0.68–1.31	
After	Mean ± SD	1.63 ± 0.86	0.48 ± 0.06	1.56 ± 0.14	17.622 (< 0.0001*)
	Median	1.52	0.52	1.58	
	Min – Max	0.69–2.68	0.40–0.53	1.38–1.71	
Test^2^ (*p* value)		2.694 (0.007*)	0.180 (0.857)	2.694 (0.007*)	

Test1: Kruskal WallisTest2: Wilcox Sign Rank test

*Statistically significant difference at p value < 0.05

**Table 5:** Three pairwise comparisons between baseline weight/hardness/surface roughness before the intervention and the wear/hardness/ surface roughness exhibited after the intervention among the three groups

Groups	Compared to	*P* value regarding wear test			*p* value regarding hardness test			*p* value regarding surface roughness test		
		Before	Before	Before		After	After		After	
CROWNTEC Saremco	BRILLIANT Crios	< 0.0001*	< 0.0001*	< 0.0001*		< 0.0001*	< 0.0001*		< 0.0001*	
	Flexcera Smile Ultra+	< 0.0001*	< 0.0001*	< 0.0001*		< 0.0001*	1.00		1.00	
BRILLIANT Crios	Flexcera Smile Ultra+	< 0.0001*	< 0.0001*	< 0.0001*		< 0.0001*	< 0.0001*		< 0.0001*	

*Statistically significant difference at *p* value < 0.05

## Discussion

Statistical analysis revealed significant differences in wear, surface roughness, and microhardness among the tested groups. Consequently, the null hypothesis was rejected.

The primary advantage of this study lies in its direct comparison between two advanced fabrication technologies, providing valuable insight into their mechanical performance under simulated oral hygiene conditions. The novelty of the work stems from the limited existing literature evaluating the long-term durability of 3D-printed restorative materials compared to conventionally milled counterparts. Clinically, these findings are highly relevant, as they offer evidence-based guidance for material selection in restorative dentistry, particularly in cases requiring enhanced wear resistance and surface integrity under routine oral care procedures.

### Wear test

The comparative assessment of wear among the tested groups, pre- and post-simulated toothbrushing, revealed an overall reduction in material weight. Notably, FSU consistently demonstrated superior wear resistance, while BC exhibited the highest degree of wear. This disparity can be largely explained by differences in filler morphology. FSU incorporates spherical silica fillers, which are known to enhance wear resistance through improved stress dispersion and reduced abrasive impact—an effect corroborated by the findings of Xing et al. [24]. In contrast, the angular morphology of BC fillers contributes to increased surface abrasion, as particle geometry significantly influences two-body wear mechanisms. Sharp-edged, angular fillers tend to cause more localized surface damage compared to smoother, spherical particles [25].

It is well-documented that wear is a complex, multifactorial process influenced by testing methodologies and conditions. Variability in test setups—including pin-on-block, pin-on-disc, three-body abrasion, and simulated toothbrushing—combined with differences in loading conditions, sliding motion, and environmental exposure (e.g., moisture or dietary substances), can lead to differing outcomes [26]. Furthermore, water sorption into the resin matrix may result in plasticization of the material or hydrolytic degradation of the silane coupling agent, both of which compromise the composite’s resistance to wear [26].

The observed post-brushing reduction in wear values for BC may be attributed to its precision-controlled fabrication as a milled CAD-CAM material. This standardized manufacturing approach likely ensured a homogenous structure, contributing to balanced wear behavior between the resin matrix and the filler particles. Conversely, the 3D-printed specimens demonstrated a more pronounced degradation of the organic matrix, leading to a rougher surface and increased material loss—underscoring the significant role of manufacturing technique in determining wear performance.

These findings diverge from those reported by Arafa et al. [27], who observed marked mass loss in both 3D-printed and milled materials. This discrepancy may be due to differences in filler content, the rigor of manufacturing protocols applied to CAD-CAM blocks (particularly under controlled thermal and pressure conditions), or the presence of an oxygen-inhibited surface layer commonly associated with 3D-printed resin composites.

### Hardness test

The comparative evaluation of microhardness before and after simulated toothbrushing revealed a general decline in Vickers Hardness Number (VHN) across all tested materials. FSU consistently exhibited the lowest hardness values, whereas BC demonstrated the highest. This suggests that the intervention effectively reduced surface hardness, albeit to varying degrees depending on the material.

Differences in surface hardness are likely influenced by compositional factors, particularly filler content and the polymerization process. The presence of inorganic fillers and the application of elevated temperatures during the fabrication of CAD/CAM resins contribute to enhanced mechanical properties, including hardness [28]. However, exposure to moisture may lead to water absorption by the resin matrix, potentially softening the material and compromising its mechanical integrity [28].

These results are consistent with those of Grzebieluch et al. [15], who reported significantly higher hardness in milled blocks due to their dense and uniform structure. A strong correlation between filler content and hardness has also been reported by Ling et al. [29] and Mirică et al. [30]. Although lower hardness is generally associated with increased susceptibility to wear [31], the present findings indicate that printed materials, despite lower hardness, showed higher wear rates, reinforcing the influence of structural integrity.

Moreover, studies by McCabe et al. [32], Son et al. [33], and Loyaga-Rendon et al. [34] further support the positive relationship between filler loading and surface hardness. In contrast, Al-Haj Husain et al. [35] found no change in hardness for 3D-printed resins post-intervention, which may be attributed to differences in polymer matrix composition.

Finally, findings from Goujat et al. [36] and Kim et al. [37] confirmed that CAD/CAM materials possess greater surface hardness than 3D-printed resins, aligning with the current results. This may be due to higher degrees of polymerization and the influence of an oxygen-inhibited layer formed prior to brushing in printed materials.

### Surface roughness test

The surface roughness analysis of CT, BC, and FSU before and after intervention revealed statistically significant differences across all groups. FSU exhibited the greatest reduction in surface roughness, followed by CT, while BC showed the least. These variations are likely due to differences in filler content and ceramic-to-polymer ratios, which influence surface characteristics of resin-based restoratives [27].

These results are consistent with Nam et al. [21], who found increased surface roughness after toothbrushing across all groups, with 3D-printed resins showing the highest roughness and milled resins the least. He attributed this to an increased surface area promoting protein adsorption through greater pentamer binding.

Similarly, Kamonkhantikul et al. [38] observed a significant increase in roughness after 20,000 toothbrushing cycles. Differences were linked to polishing protocols, filler size and shape, brush design, bristle type, and toothpaste abrasivity.

Siddanna et al. [39] noted that surface roughness is influenced by filler type, size, form, and content. Even materials fabricated with identical milling instruments showed different roughness due to variations in resin matrix properties and milling strategies [40]. Although BC and Cerasmart (CS) share similar compositions, they exhibited different roughness levels—attributed to CS’s smaller, rounder fillers versus BC’s angular fillers [39].

Flury et al. [41] also reported significant surface roughness increase post-brushing, despite using a low-abrasivity toothpaste (70 RDA). This was due to the mechanical action of brushing and simulating intraoral wear. Ellakany et al. [42] further supported this, suggesting that the absence of diamond polishing paste following standard polishing could lead to rougher surfaces. Future studies should investigate the effect of diamond paste application versus glazing on surface finish.

### Limitations

The experimental toothbrushing protocol in this study did not fully replicate key aspects of the oral environment, such as the dynamic fluctuations in pH, presence of salivary enzymes and microbial biofilms, or the mechanical forces generated during mastication. The absence of these critical biological and mechanical factors limits the extrapolation of in vitro results to clinical performance. Additionally, the study utilized flat, standardized specimens lacking anatomical contours, which do not accurately represent the complex curvature and surface topography of natural teeth. Incorporating more physiologically relevant conditions—such as cyclic loading to mimic chewing forces, temperature changes, and the presence of saliva and oral microbiota—would enhance the clinical relevance and predictive value of future wear simulations.

## Conclusion

Based on the findings of this study, it was concluded that:

1. Each material was affected by artificial toothbrushing.
2. FSU consistently showed the lowest wear, CT the moderate and BC the highest.
3. The highest value in hardness was shown in BC and the lowest value was presented in FSU.
4. Milled resin demonstrated the lowest surface roughness. On the other hand, 3D printed resin exhibited the highest surface roughness.

## Recommendation

Therefore, therapeutic usage of 3D printing technology will boost productivity and make permanent restorative fabrication easier. For widespread use of 3D printing in dentistry, more research is needed to assess compressive, tensile, shear, fatigue, solubility, and permeability. Future studies should examine the physical properties of 3D-printed resin materials. The future requires standardized techniques and studies that better reflect the oral environment.

## Data Availability

No datasets were generated or analysed during the current study.

## Abbreviations

CT: CROWNTEC Saremco permanent resin
FSU: Flexcera Smile Ultra+
BC: CAD-CAM BRILLIANT Crios
CLSM: Confocal laser scanning microscopy
VHN: Vickers Hardness Number
DLP: Digital Light Processing

## References

[1] Sasany R, Jamjoon FZ, Kendirci MY, Yilmaz B. Effect of printing layer thickness on optical properties and surface roughness of 3D-printed resins: an in vitro study. Int J Prosthodont. 2024;37:165–73. 10.11607/ijp.8965. 38787581 10.11607/ijp.8965

[2] Jovanović M, Živić M, Milosavljević M. A potential application of materials based on a polymer and CAD/CAM composite resins in prosthetic dentistry. J Prosthodont Res. 2021;65:137–47. 10.2186/jpr.JPOR_2019_404. 32981910 10.2186/jpr.JPOR_2019_404

[3] Bompolaki D, Lubisich EB, Fugolin AP. Resin-based composites for direct and indirect restorations: clinical applications, recent advances, and future trends. Dent Clin North Am. 2022;66:517–36. 10.1016/j.cden.2022.05.003. 36216444 10.1016/j.cden.2022.05.003

[4] Wu X, Mu F, Lin Z. Three-dimensional printing of graphene-based materials and the application in energy storage. Mater Today Adv. 2021;11:100157. 10.1016/j.mtadv.2021.100157.

[5] Barot T, Rawtani D, Kulkarni P. Nanotechnology-based materials as emerging trends for dental applications. Rev ADV Mater Sci. 2021;60:173–89. 10.1515/rams-2020-0052.

[6] Sasany R, Donmez MB, de Paula MS, Kahveci Ç, Ceylan G, Yilmaz B, et al. Stainability and translucency of potassium aluminum sulfate applied computer-aided design and computer-aided manufacturing materials after coffee thermocycling. J Esthet Restor Dent. 2024;36:477–83. 10.1111/jerd.13154. 37877244 10.1111/jerd.13154

[7] Branco AC, Colaço R, Figueiredo-Pina CG, Serro AP. Recent advances on 3D-printed zirconia-based dental materials: a review. Mater (Basel). 2023;16:1860. 10.3390/ma16051860. 10.3390/ma16051860 PMC10004380 36902976

[8] Marchesi G, Camurri Piloni A, Nicolin V, Turco G, Di Lenarda R. Chairside CAD/CAM materials: current trends of clinical uses. Biology (Basel). 2021;10:1170. 10.3390/biology10111170. 34827163 10.3390/biology10111170 PMC8614873

[9] Teng X, Li F, Lu C. Visualization of materials using the confocal laser scanning microscopy technique. Chem Soc Rev. 2020;49:2408–25. 10.1039/c8cs00061a. 32134417 10.1039/c8cs00061a

[10] Van Meter KE, Krick BA. Friction and wear testing. Characterization and failure analysis of plastics. ASM International; 2022;341 – 52. 10.31399/asm.hb.v11b.a0006911.

[11] Zaim B, Serin Kalay T, Purcek G. Friction and wear behavior of chairside CAD-CAM materials against different types of antagonists: an in vitro study. J Prosthet Dent. 2022;128:803–13. 10.1016/j.prosdent.2021.09.024. 34823868 10.1016/j.prosdent.2021.09.024

[12] de Andrade GS, Augusto MG, Simões BV, Pagani C, Saavedra G, Bresciani E. Impact of simulated toothbrushing on surface properties of chairside CAD-CAM materials: an in vitro study. J Prosthet Dent. 2021;125:469.e461-469.e466. 10.1016/j.prosdent.2020.08.028. 10.1016/j.prosdent.2020.08.028 33279154

[13] Baloch H. Application of confocal laser scanning microscopy in dentistry. J Adv Microsc Res. 2018;9:245–52. 10.1166/jamr.2014.1217.

[14] Weller D, Colombier M, Cáceres F, Vasseur J, Dingwell DB, Scheu B. Confocal Scanning Laser Microscopic (CSLM) characterization of volcanic rocks. J Volcanol Geotherm Res. 2024;446:107992. 10.1016/j.jvolgeores.2023.107992.

[15] Grzebieluch W, Kowalewski P, Grygier D, Rutkowska-Gorczyca M, Kozakiewicz M, Jurczyszyn K. Printable and machinable dental restorative composites for CAD/CAM application-comparison of mechanical properties, fractographic, texture and fractal dimension analysis. Mater (Basel). 2021;14:4919. 10.3390/ma14174919. 10.3390/ma14174919 PMC8434230 34501009

[16] Bandyopadhyay A, Mitra I, Avila JD, Upadhyayula M, Bose S. Porous metal implants: processing, properties, and challenges. Int J Extrem Manuf. 2023;5:032014. 10.1088/2631-7990/acdd35. 37476350 10.1088/2631-7990/acdd35 PMC10355163

[17] Akgungor G, Sen D, Aydin M. Influence of different surface treatments on the short-term bond strength and durability between a zirconia post and a composite resin core material. J Prosthet Dent. 2008;99:388–99. 10.1016/s0022-3913(08)60088-8. 18456050 10.1016/S0022-3913(08)60088-8

[18] Turker I, Kursoglu P. Wear evaluation of CAD-CAM dental ceramic materials by chewing simulation. J Adv Prosthodont. 2021;13:281–91. 10.4047/jap.2021.13.5.281. 34777718 10.4047/jap.2021.13.5.281 PMC8558571

[19] El Sayed SM, Basheer RR, Bahgat SFA. Color stability and fracture resistance of laminate veneers using different restorative materials and techniques. Egypt dent J. 2016;62:1–15.

[20] Teixeira EC, Thompson JL, Piascik JR, Thompson JY. In vitro toothbrush-dentifrice abrasion of two restorative composites. J Esthet Restor Dent. 2005;17:172–80. 10.1111/j.1708-8240.2005.tb00109.x. 15996389 10.1111/j.1708-8240.2005.tb00109.x

[21] Nam NE, Hwangbo NK, Kim JE. Effects of surface glazing on the mechanical and biological properties of 3D printed permanent dental resin materials. J Prosthodont Res. 2024;68:273–82. 10.2186/jpr.JPR_D_22_00261. 37245959 10.2186/jpr.JPR_D_22_00261

[22] Wang L, Garcia FCP, De Araújo PA, Franco EB, Mondelli RFL. Wear resistance of packable resin composites after simulated toothbrushing test. J Esthet Restor Dent. 2004;16:303–14. 10.1111/j.1708-8240.2004.tb00058.x. 15726799 10.1111/j.1708-8240.2004.tb00058.x

[23] Rashid H. Comparing glazed and polished ceramic surfaces using confocal laser scanning microscopy. J Adv Microsc Res. 2012;7:208–13. 10.1166/jamr.2012.1117.

[24] Xing X, Li R. Wear behavior of epoxy matrix composites filled with uniform sized sub-micron spherical silica particles. Wear. 2004;256:21–6. 10.1016/S0043-1648(03)00220-5.

[25] Tsujimoto A, Barkmeier WW, Fischer NG, Nojiri K, Nagura Y, Takamizawa T, et al. Wear of resin composites: current insights into underlying mechanisms, evaluation methods and influential factors. Jpn Dent Sci Rev. 2018;54:76–87. 10.1016/j.jdsr.2017.11.002. 29755618 10.1016/j.jdsr.2017.11.002 PMC5944074

[26] Kessler A, Reymus M, Hickel R, Kunzelmann KH. Three-body wear of 3D printed temporary materials. Dent Mater. 2019;35:1805–12. 10.1016/j.dental.2019.10.005. 31727446 10.1016/j.dental.2019.10.005

[27] Arafa AM, Ghanem L. Wear and surface roughness of 3D printed and milled CAD-CAM restorative materials. Al-Azhar J Dent Sci. 2023;26:147–59. 10.21608/AJDSM.2023.186911.1406.

[28] Temizci T, Bozoğulları HN. Effect of thermocycling on the mechanical properties of permanent composite-based CAD-CAM restorative materials produced by additive and subtractive manufacturing techniques. BMC Oral Health. 2024;24:334. 10.1186/s12903-024-04016-z. 38486195 10.1186/s12903-024-04016-z PMC10938812

[29] Ling L, Ma Y, Malyala R. A novel CAD/CAM resin composite block with high mechanical properties. Dent Mater. 2021;37:1150–5. 10.1016/j.dental.2021.03.006. 33849756 10.1016/j.dental.2021.03.006

[30] Mirică IC, Furtos G, Bâldea B, Lucaciu O, Ilea A, Moldovan M, et al. Influence of filler loading on the mechanical properties of flowable resin composites. Mater (Basel). 2020;13:1477. 10.3390/ma13061477. 10.3390/ma13061477 PMC7142558 32213949

[31] Mandikos MN, McGivney GP, Davis E, Bush PJ, Carter JM. A comparison of the wear resistance and hardness of indirect composite resins. J Prosthet Dent. 2001;85:386–95. 10.1067/mpr.2001.114267. 11319537 10.1067/mpr.2001.114267

[32] McCabe JF, Walls AW, editors. Applied dental materials. Wiley; 2013.

[33] Son SA, Park JK, Seo DG, Ko CC, Kwon YH. How light attenuation and filler content affect the microhardness and polymerization shrinkage and translucency of bulk-fill composites? Clin Oral Investig. 2017;21:559–65. 10.1007/s00784-016-1920-2. 27475636 10.1007/s00784-016-1920-2

[34] Loyaga-Rendon PG, Takahashi H, Hayakawa I, Iwasaki N. Compositional characteristics and hardness of acrylic and composite resin artificial teeth. J Prosthet Dent. 2007;98:141–9. 10.1016/s0022-3913(07)60047-x. 17692595 10.1016/S0022-3913(07)60047-X

[35] Al-Haj Husain N, Feilzer AJ, Kleverlaan CJ, Abou-Ayash S, Özcan M. Effect of hydrothermal aging on the microhardness of high- and low-viscosity conventional and additively manufactured polymers. J Prosthet Dent. 2022;128:822e821. 10.1016/j.prosdent.2022.08.022. 10.1016/j.prosdent.2022.08.022 36202632

[36] Goujat A, Abouelleil H, Colon P, Jeannin C, Pradelle N, Seux D, et al. Mechanical properties and internal fit of 4 CAD-CAM block materials. J Prosthet Dent. 2018;119:384–9. 10.1016/j.prosdent.2017.03.001. 28552287 10.1016/j.prosdent.2017.03.001

[37] Kim GT, Go HB, Yu JH, Yang SY, Kim KM, Choi SH, et al. Cytotoxicity, colour stability and dimensional accuracy of 3D printing resin with three different photoinitiators. Polymers. 2022;14:979. 10.3390/polym14050979. 35267799 10.3390/polym14050979 PMC8912826

[38] Kamonkhantikul K, Arksornnukit M, Lauvahutanon S, Takahashi H. Toothbrushing alters the surface roughness and gloss of composite resin CAD/CAM blocks. Dent Mater J. 2016;35:225–32. 10.4012/dmj.2015-228. 27041012 10.4012/dmj.2015-228

[39] Siddanna GD, Valcanaia AJ, Fierro PH, Neiva GF, Fasbinder DJ. Surface evaluation of resilient CAD/CAM ceramics after contouring and polishing. J Esthet Restor Dent. 2021;33:750–63. 10.1111/jerd.12735. 33973352 10.1111/jerd.12735

[40] Kou W, Molin M, Sjögren G. Surface roughness of five different dental ceramic core materials after grinding and polishing. J Oral Rehabil. 2006;33:117–24. 10.1111/j.1365-2842.2006.01546.x. 16457671 10.1111/j.1365-2842.2006.01546.x

[41] Flury S, Diebold E, Peutzfeldt A, Lussi A. Effect of artificial toothbrushing and water storage on the surface roughness and micromechanical properties of tooth-colored CAD-CAM materials. J Prosthet Dent. 2017;117:767–74. 10.1016/j.prosdent.2016.08.034. 27836147 10.1016/j.prosdent.2016.08.034

[42] Ellakany P, Madi M, Aly NM, Alshehri T, Alameer ST, Al-Harbi FA. Influences of different CAD/CAM ceramic compositions and thicknesses on the mechanical properties of ceramic restorations: an in vitro study. Mater (Basel). 2023;16. 10.3390/ma16020646. 10.3390/ma16020646 PMC9865408 36676383

